# False-positivity in diagnosis of brucellosis associated with Rev-1 vaccine

**DOI:** 10.3402/ljm.v8i0.20417

**Published:** 2013-02-14

**Authors:** Hayati Gunes, Mustafa Dogan

**Affiliations:** 1 Department of Medical Microbiology, Medical Faculty, Namik Kemal University, Tekirdağ, Turkey; 2 Department of Infectious Disease, Medical Faculty, Namik Kemal University, Tekirdağ, Turkey

Brucellosis is a zoonotic disease caused by *Brucella* spp. and is still a serious problem of public health for some regions around the world such as the Mediterranean Sea, Middle East, Latin America, and Asia (1).

These pathogenic bacteria can infect humans as well as sheep, goats, etc. *B. melitensis* (sheep and goats) is the most important causative agent for human brucellosis and is followed by *B. ovis* (sheep), *B. abortus* (cattle), *B. suis* (pigs), and *B. canis* (dogs). *Brucella* causes an important disease characterized by decreased fertility in rams, sporadic abortions in sheep and increased lamb mortality. Control strategies to prevent human brucellosis include pasteurization of milk, livestock vaccination and elimination of infected animals. In animal populations, mass vaccination accompanied by a strict surveillance scheme is a first step to reduce the number of infected animals (2). A test-and-slaughter program can be applied in order to obtain brucellosis-free flocks and regions (3). Despite the fact that subcutaneous vaccinations can create interferences with the serological diagnosis of brucellosis and that this strain can be pathogenic for humans (4), the live attenuated *Brucella melitensis* Rev-1 vaccine is considered to be the best vaccine available for the control of sheep and goat brucellosis (3, 4). This vaccine has variable protective efficacy ranging between 40 and 100% because of the stringency of the challenge and other factors (5).

Agri is a city in eastern Turkey with a population >500,000 in the city center, districts, and villages. The people live on agriculture and animal husbandry.

The Provincial Agricultural Directorate performed a vaccine campaign for the prevention of brucellosis in sheep in Agri. Rev-1 vaccine was administered subcutaneously to 300,000 sheep and a total of 46 individuals – including 33 veterinarians and 13 veterinary technicians – commissioned in this campaign.

After finishing the campaign, Brucella serological tests of the assigned staff were examined. Rose-Bengal test was positive in 10 of them. The Wright agglutination tests were applied to the sera for confirmation of Rose-Bengal test results. The titer was 1/80 in one of them and positive in the remaining, i.e. 1/160 and higher. The treatment was started for two of these individuals with prominent clinical findings. One of them stopped receiving the treatment after 2 weeks, and the other received treatment for 6 weeks. This test was repeated after 2 weeks and all of the Brucella tube agglutination titers were determined as 1/80 and below.

Brucellosis has grave economic impacts in the local population and can also cause serious problems in the national agricultural economy. Since slaughtering is the only solution when an animal is infected, *Brucella* vaccination in animals has gained great significance. Starting the treatment as a result of false-positivity leads not only to increased treatment costs but also to unwanted side effects. Repeating the tests adds costs and takes time that may delay treatment. In a recent study (6), the modified Rose-Bengal test showed lower values from 1/4 dilutions in such individuals. Therefore, we conclude that after the Rev-1 vaccine, we can observe false-positivity of *Brucella* Rose-Bengal test. In this case, the modified Rose-Bengal test should be applied with the lower values from 1/4 considered as negative.

*Hayati Gunes* Department of Medical Microbiology Medical Faculty Namik Kemal University Tekirdağ, Turkey Email: dr_hgunes@yahoo.com *Mustafa Dogan* Department of Infectious Disease Medical Faculty Namik Kemal University Tekirdağ, Turkey
